# Inhibition of HIV and HSV infection by vaginal lactobacilli *in vitro* and *in vivo*

**DOI:** 10.1186/2008-2231-20-53

**Published:** 2012-10-15

**Authors:** Rezvan Zabihollahi, Elahe Motevaseli, Seyed Mehdi Sadat, Ali Reza Azizi-Saraji, Sogol Asaadi-Dalaie, Mohammad Hossein Modarressi

**Affiliations:** 1 Hepatitis and AIDS department, Pasteur institute of Iran, Tehran, Iran; 2 School of Medicine, Tehran University of Medical Sciences (TUMS), Tehran, Iran

**Keywords:** HIV, HSV, Sexually transmitted disease, Lactobacillus

## Abstract

**Background and the purpose of the study:**

The cervico-vaginal mucosa which is populated with microflora (mostly includes lactobacilli) is the portal of entry for sexually transmitted pathogens.

**Methods:**

The *in vitro* anti-viral effect of vaginal and non-vaginal lactobacillus was evaluated using single cycle HIV-1 replication and HSV-2 plaque reduction assays. The XTT proliferation assay was used to monitor the cellular toxicity. The *in vivo* anti-HSV-1 activity was evaluated in BALB/c mouse model by monitoring skin lesion and immune response development.

**Results and major conclusion:**

DMEM culture supernatant of *L. Gasseri* and *L. fermentum* (PH 7.3) did not show toxic effect but inhibited 50% of HIV replication at 12 and 31% concentrations, respectively. Co-culture of *L. gasseri* (1000 CFU/ target cell) showed mild cytotoxicity but inhibited 68% of HIV replication. The supernatant of *L. crispatus* inhibited 50% of HSV replication at 4% and also co-culture of *L. gasseri, L. rhamnosus* and *L. crispatus* revokes almost all of the HSV multiplication. Culture supernatants of *L. gasseri* and *L. crispatus* had significant virucidal effect against the HIV and HSV and inhibited HSV infection in a stage before viral entry to the target cells. Alive *L. gasseri* cells showed high potential for inhibiting HSV-1 infection *in vivo* condition. Current data indicates that lactobacilli supernatant encompasses components with neutralizing activity against HIV and HSV and it would be a determinant factor for viral diseases transmission and promising lead for anti-viral probiotic design.

## Introduction

The cervico-vaginal mucosa is the portal of entry for sexually transmitted pathogenic microorganisms. In child-bearing age healthy females, the vaginal protective mucosa is populated with microflora that mostly includes lactobacilli. Vaginal health is positively associated with dominance of lactobacilli over pathogenic anaerobes [1]. Bacterial vaginosis (BV) is a situation with an imbalance of the vaginal microbial flora and is not caused by specific pathogenic microorganisms [2]. Reduction, absence or lack of antimicrobial properties (production of acid and H_2_O_2_) of lactobacilli and their replacement by anaerobic microbes such as *Gardnerella vaginalis* may be seen in BV [3].

Increasing data indicate that lacking lactobacilli or abnormal vaginal flora facilitates the transmission of viral sexually transmitted diseases (STD) [4]. There are some data reporting that HIV seropositivity correlates with BV independent of other behavior variabilities [5,6]. Recent prospective investigations demonstrated the association between vaginal flora alterations and the acquisition of Human immunodeficiency virus (HIV) infection [7]. Also, reduction in vaginal flora lactobacillus content was identified as a predisposing factor for infection of human papillomavirus (HPV) and herpes simplex virus type (HSV) [8-10]. Recent studies have revealed that abnormal vaginal flora triggers the shedding of HSV and cytomegalovirus (CMV) in women genital tract [11,12]. On the other hand genital herpes infection is a major risk factor for acquisition and transmission of HIV via sexual contact [13,14]. Moreover, HIV copy number in female genital-tract discharge inversely correlates with lactobacilli counts in bacterial flora [15].

Therefore lactobacilli exhibit an important role for viral infections in both the female health protection as well as reducing the risk of infection transmission to a healthy man. *In vitro* studies have disclosed that hydrogen peroxide production by *L. acidophilus* strain displays a virucidal effect against HIV-1 [16]. Additionally, it has been reported that pre-incubation with different lactobacillus strains reduces the infection titer of vesicular stomatitis virus (VSV) [17]. However the inhibition mechanism of lactobacilli against viral infections is poorly understood, but nevertheless metabolic products such as lactic acid, H_2_O_2_ and bacteriocins are possible mediators to account for the protection against viruses. These factors might act against viral particles by inactivation, epithelial cell attachment competition, mucin gel preservation or maintaining the appropriate innate immune response [18-20].

In spite of the clinical and epidemiological studies, the *in vitro* anti-viral activity of these probiotic bacteria in cell culture environment has not been investigated in detail. The purpose of this study is to evaluate anti-HIV and HSV activity of vaginal lactobacilli *in vitro* and *in vivo* conditions and determine the possible mode of action.

## Materials and methods

### Lactobacilli strains and growth condition

Vaginal lactobacilli strains; *L. crispatus ATCC33820*, *L. gasseri ATCC33323, L. rhamnosus GG* and *L. fermentum ATCC14931* were used in this study as well as the non-vaginal ones; *L. acidophilus LA-5* (Hansen company, USA), *L. paracasei subsp. Casei ATCC25302*, *L. acidophilus NCFM* and *L. casei CRL431* (Hansen company, USA). All bacteria were stored at −70°C in 85% de Man- Rogosa-Sharpe (MRS) supplemented with 15% glycerol. Lactobacilli were inoculated from frozen glycerol vials onto MRS broth and incubated at 37°C for 48hs under anaerobic conditions [21]. Plating of serial lactobacilli 10-fold dilutions in MRS-agar was used to determine the colony forming units (CFU) titer. Colony counting was carried out after 48hs of incubation.

### Culture supernatant

Lactobacilli were harvested from fresh MRS culture by centrifuging at 3 × 10^3^ g and the pellet was washed with DMEM twice to eliminate the remnants MRS. Working stokes were prepared in DMEM (2 × 10^9^ CFU/ml) and stored at 4°C. These working stokes were freshly (no more than 2 weeks) used for experiments. To prepare the culture supernatant (CS), lactobacilli (3.6 × 10^8^ CFU) were inoculated onto each well of 6-wells plates containing 6 ml of high glucose DMEM and incubated for 20hs in a CO_2_ incubator. Culture supernatant (neutral pH phase) was harvested by clarification of the medium with 0.22 μm filters and used freshly for each experiment.

### Cell culture and transfection

HEK293T (Human emberionic kidney), Hela (Cervix cancer) and vero (African green monkey kidney) cell lines were cultured in Dulbecco’s Modified Eagle Medium (DMEM, Gibco, USA) supplemented with 10% fetal bovine serum (FBS, Gibco, USA), 5 mM glutamine. The cells medium was supplemented with penicillin (100 IU/ml) and streptomycin (100 μg/ml) in particular experiments. HEK293T cells were transfected using polyfect transfection reagent (Qiagen, USA). Transfection was performed according to the manufacturer’s protocol in 6-wells plates [22].

### Viruses

Single cycle replicable (SCR) HIV-1 (NL4-3) and HSV-2 viruses were used in this study. Plasmid mixture (2 μg) of PmzNL4-3 [23], pSPAX.2 and pMD2G was cotransfected into the HEK293T cells to prepare VSV surface glycoprotein (VSVG) pseudotyped SCR HIV-1 [24]. Virus containing supernatant was harvested and pooled 24, 48 and 72hs post transfection.

Vero cells were infected to supply the HSV-2 virus stokes. The Cells were seeded onto 6-wells plates (4.5 × 10^5^ cells/well) and incubated for one day to reach 98% of confluence. One milliliter of virus supernatant was used to infect cells in each well and unbound virions were removed after 2hs. Virus containing supernatants were harvested and pooled every 24hs after infection until day 4.

HIV and HSV virus containing supernatants were clarified using 0.22 μm filters and 10mins centrifuging at 10^4^ g. Viruses were stored at −70°C and assessed for infectious titer using replication and plaque reduction assays.

### HIV replication assay

Single cycle replication assay was used to evaluate the inhibitory activity against replication of VSVG psudotyped SCR HIV-1 virions [24,25]. Hela cells were seeded in 96-wells plates (6 × 10^3^ cells/well) and maintained for 24hs in antibiotic free medium. Lactobacilli cells or supernatant were added into the wells 2hs before infection. Cells were infected with SCR HIV-1 virions (600 ng P24) and incubated for 20hs to accelerate the virions adsorption [22]. After virus entry, the cells were washed two times with DMEM to remove unbound viral particles. Cells were fed with antibiotic containing medium and incubated for additional 48 hrs. Plates were centrifuged for 15mins at 3 × 10^3^ g and P24 content of the cells supernatant was evaluated using P24 capture ELISA (Biomerieux, France).

### HSV plaque reduction assay

The inhibitory effect of lactobacilli against multiplication of HSV was investigated using plaque reduction assay. Vero cells were placed into the 24-wells culture plates (Nunc, Denmark) at density of 4 × 10^5^ cells per well and incubated for 24hs (in antibiotic free medium) to reach at least 98% of confluence. The cell monolayer was infected with 50pfu of HSV and washed after 1hs to remove unbound virions. The cell monolayer were then overlaid with DMEM supplemented by 1.5% of methylcellulose, 5% FBS and antibiotics. After 72hs, the overlay medium was removed and the cells were washed twice with DMEM and fixed by methanol. The formed plaques were counted after staining with 0.5% crystal violet.

### Time-of-addition study

The inhibitory effect of *L. gasseri* and *L. crispatus* supernatant against different intervals of HSV replication was examined according to the previously described procedure with some minor modifications [26]. Vero cells monolayer was prepared by seeding the cells into 24-well plates (Nunc, Denmark) as describe above. Then, 20% concentration of *L. gasseri* and *L. crispatus* CS were added into the cells environment before (−6 and −2 hs), during (0 hs) or after (2 and 6 hs) time course of HSV-2 infection. Afterwards, the procedures similar to plaque reduction assay section were performed except that cell monolayer was washed twice by DMEM to eliminate the lactobacilli supernatant in pre-infection (−6, −2 and 0hs) groups. In other groups the lactobacilli CS was added after infection (2 and 6hs) and the concentration was kept constant until the end of the test.

### Virucidal assay

The direct effect of *L. gasseri* and *L. crispatus* CS on HSV and HIV infectivity was evaluated by using virucidal assay. Different concentrations (2, 20 and 50%) of CSs were mixed thoroughly with HSV (5 × 10^3^ pfu) and HIV (1.2 × 10^3^ P24) virions, in final volume of 10 μl. The mixtures were incubated at 37°C for 2hs and then the fresh medium (90 μl) was added to each tube. Residual virus infectivity was determined by plaque reduction and HIV replication assays as described above.

### In vivo anti-HSV assay

Animal consisted of pathogen-free, female BALB/c mice with 6–8 weeks of age-average 20 g of weight (purchased from Pasteur Institute of Iran, Iran) were handled according to the guidelines of the national institute of health guide and care for use of laboratory animal, Iran. The skin of BALB/c mice was shaved in right flank and scratched by needle (gauge 22). The naked and scratched skin was infected with 10^4^ pfu of HSV-1 virions. Alive lactobacilli suspension (10^5^ CFU in 20 μl of PBS) was added immediately after infection. Mice were maintained for six weeks and then evaluated for skin lesions and weight loss. The acyclovir cream (5%) was used as positive control. The immune response against HSV was also monitored by extracting the spleen cells and investigating their proliferative response to HSV antigens. Total spleen cells were treated with lysis buffer to remove red blood cells. Cell suspension was adjusted to 5.1 × 10^6^ cells per milliliter and cultured (120 μl) in each well of 96-well plates with 10^3^pfu of inactivated HSV for 72hs. Phytohemagglutinin-A (PHA 7 μg/ml; Gibco, USA) was used as control. The proliferation of the cells was measured by addition of 50 μl of XTT (sodium 3_-[1-(phenylaminocarbonyl)- 3,4-tetrazolium]-bis(4-methoxy-6-nitro)benzene sulfonic acid; Roche, Germany) into each well. The plates were incubated at 37°C for 2hs and then read at test and references wavelengths of 450 and 630 nm, respectively.

### Cytotxicity assay

The toxicity of lactobacilli cells and supernatant for Vero and Hela cells was determined using XTT (Roche, Germany) proliferation assay as previously is described. Vero cells were cultured in 96-wells plates (6 × 10^3^/well) (Nunc, Denmark) contain lactobacilli cells (6 × 10^7^ CFU/ml) and CS. Bacterial cells were removed after 1 hr and antibiotic containing medium was added into the wells. After 72hs, the medium was discarded and the cells were subsequently rinsed with phosphate buffered saline (PBS). To study the effect of lactobacilli cells and supernatant on Hela cells viability, HIV replication assay plates were subjected to the proliferation assay directly after washing cells with PBS. The XTT reagent (40 μl/well) was added into the plates and incubated at 37°C for additional 3hs. The optical densities (OD) were measured using enzyme immunoassay reader (stat fax2100, Awareness, USA) at test and reference wavelengths of 450 nm and 630 nm respectively.

## Results and discussion

### Anti-viral activity of lactobacilli supernatant

The DMEM culture supernatant (CS) of lactobacilli (pH of 7.3) was used to appraise the overall anti-HIV and HSV activity (Table 1). Virus multiplication was significantly decreased by all lactobacilli CS, although to different extents. *L. gasseri, L. fermentum, L. acidophilus* and *L. crispatus* inhibited 50% of HIV-1 virions replication at 12, 31, 46 and 48% concentration, respectively. Parallel experiments were performed to investigate the anti-HSV-2 activity of lactobacilli CS using plaque reduction assay. Lactobacilli CSs showed very high activity for inhibition of HSV virions replication. *L. crispatus* CS inhibited plaques formation by 50% in the very low concentration (4%) that reveals noteworthy anti-HSV activity of this strain. Other lactobacilli also showed moderate to strong anti-HSV activity. *L. gasseri, L. acidophilus,L. rhamnosus* and *L. casei* inhibited the HSV replication with IC_50_ value of 11, 14, 17 and 19%. *L. crispatus* showed significant inhibitory activity for HSV but was not effective for inhibition of HIV replication. This finding emphasizes the anti-HSV active components of this strain inhibit HSV in a specific manner.

**Table 1:** The antiviral activity of lactobacilli supernatant

**Lactobacilli strain**	**Antiviral activity (%)**			
	**HIV-1 Inhibitory**		**HSV-2 Inhibitory**	
	**IC**_**50**_**(v/v)**	**CC**_**50**_**(v/v)**	**IC**_**50**_**(v/v)**	**CC**_**50**_**(v/v)**
*L. crispatus*	48.5 ± 4.4	NT	4.2 ± 1.7	NT
*L. acidophilus (Hansen co.)*	46.3 ± 8.3	NT	>50	NT
*L. paracasei subsp. Casei*	≥50	NT	43.1 ± 3.6	NT
*L. rhamnosus*	≥50	NT	17.7 ± 6.3	NT
*L. fermentum*	31.7 ± 4.1	≥50	>50	≥50
*L. casei (Hansen co.)*	≥50	NT	19.3 ± 1.6	NT
*L. gasseri*	12.2 ± 3.5	47.4 ± 2.1	11.2 ± 3.3	≥50
*L. acidophilus*	≥50	NT	14.9 ± 5.2	NT

IC_50_ is a concentration (v/v) which is able to inhibit 50% of virus multiplication; CC_50_ is a concentration (v/v) which is 50% toxic for target cells; *NT*, Not toxic.

The cytotoxicity results showed that the CS from vaginal and non-vaginal lactobacilli had no toxicity for target cells. This indicates the safety of these strains and selective activity of their CS for reducing of viral replication rather than cellular proliferation.

Moderate anti-HIV and strong anti-HSV activity of the lactobacilli supernatants demonstrate that the lactobacilli CS encompass molecules with considerable specific activity for inhibition of viral replication. These data also revealed notably difference between the anti-viral activity of vaginal and non-vaginal strains (Table 1). The anti-viral activity of lactobacilli due to disinfectant activity of MRS culture supernatants was previously reported [16,27]. Although in this study the neutral pH lactobacilli supernatant of lactobacilli in DMEM showed no cytotoxicity but significant anti-viral activity implying the presence of active compounds rather than disinfectant activity.

### The anti-viral activity of co-cultured lactobacilli

**Figure 1:**
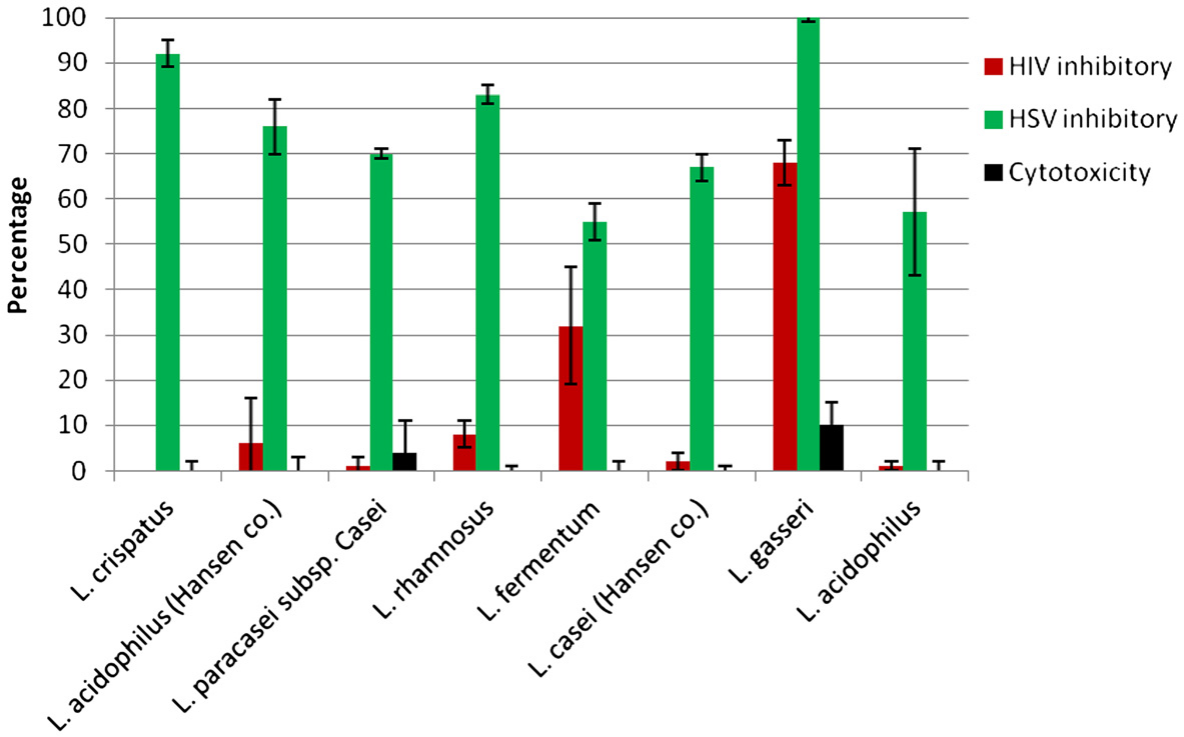
**The anti-viral potential of lactobacilli co-culture against HSV-2 and HIV infection in co-cultures.** Lactobacilli were added to the cells environment one hour before infection and removed by washing after viral adhesion to the cells. Cells were fed with antibiotic containing medium after infection.

The potential of lactobacilli for inhibition of HSV-2 and HIV-1 replication was studied by co-culturing the lactobacilli with cells. Lactobacilli were added into the cells environment considering physiological concentration in vaginal environment (100 CFU/cell) one hour before infection and removed by washing after viral adhesion to the cells. Antibiotics were added to the medium after infection to eradicate any growth of bacteria after viral entry. The cytotoxicity of lactobacilli was investigated in a parallel experiment. The vaginal lactobacilli strains (*L. gasseri*, *L. crispatus* and *L. rhamnosus*) showed higher potential for inhibition of HSV virion (Figure 1). *L. gasseri* blocked almost all of HSV virions infection however *L. crispatus* and *L. rhamnosus* inhibited this virus by 93 and 84 percent, respectively. Approximately 68 and 31% of HIV inhibition was observed by *L. gasseri* and *L. fermentum* co-culture, whereas little inhibition was shown by other lactobacilli strains investigated in this study. Our results demonstrated that presence of vaginal lactobacilli significantly reduces viral entry to the target cells although the non-vaginal ones were apparently lesser potent. All of investigated vaginal strains were active for blocking HSV however only two strains (*L. gasseri* and *L. fermentum*) had anti-HIV activity.

### Mode of anti-viral activity

Acording to the virucidal assay *L. crispatus* and *L. gasseri* inactivated 74 and 24%of HSV virions in 50% concentration, respectively (Table 2). The remarkable virucidal activity of lactobacilli CS is in consistent with the protective effect of lactobacilli against HSV and HIV virion in co-culture condition (Figure 1), suggesting that lactobacilli may secrete bacteriocins or other sort of molecules with viral neutralization activity. This mode of anti-viral activity is rather different from previously reported ones (due to H_2_O_2_ and H^+^ secretion) [27] since CS used in this study was in neutral pH and had no toxic effect for target cells.

**Table 2:** The virucidal effect of lactobacilli supernatant

**Lactobacillus Strain**	**HIV virucidal effect (%)**			**HSV-2 virucidal effect (%)**		
	**2%v/v**	**20%v/v**	**50%v/v**	**2%v/v**	**20%v/v**	**50%v/v**
*L. crispatus*	1.1 ± 0.5	8.5 ± 0.6	14.3 ± 3.3	15.6 ± 8.7	28.6 ± 3.9	76.4 ± 7.2
*L. gasseri*	4.3 ± 1.7	7.3 ± 1.5	15.4 ± 1.4	0.7 ± 0.2	6.3 ± 1.7	24.7 ± 3.2
*L. rhamnosus*	0.1 ± 0.02	2.1 ± 0.09	11.8 ± 0.01	4.2 ± 0.9	15.6 ± 2.1	38.3 ± 4.5

**Figure 2:**
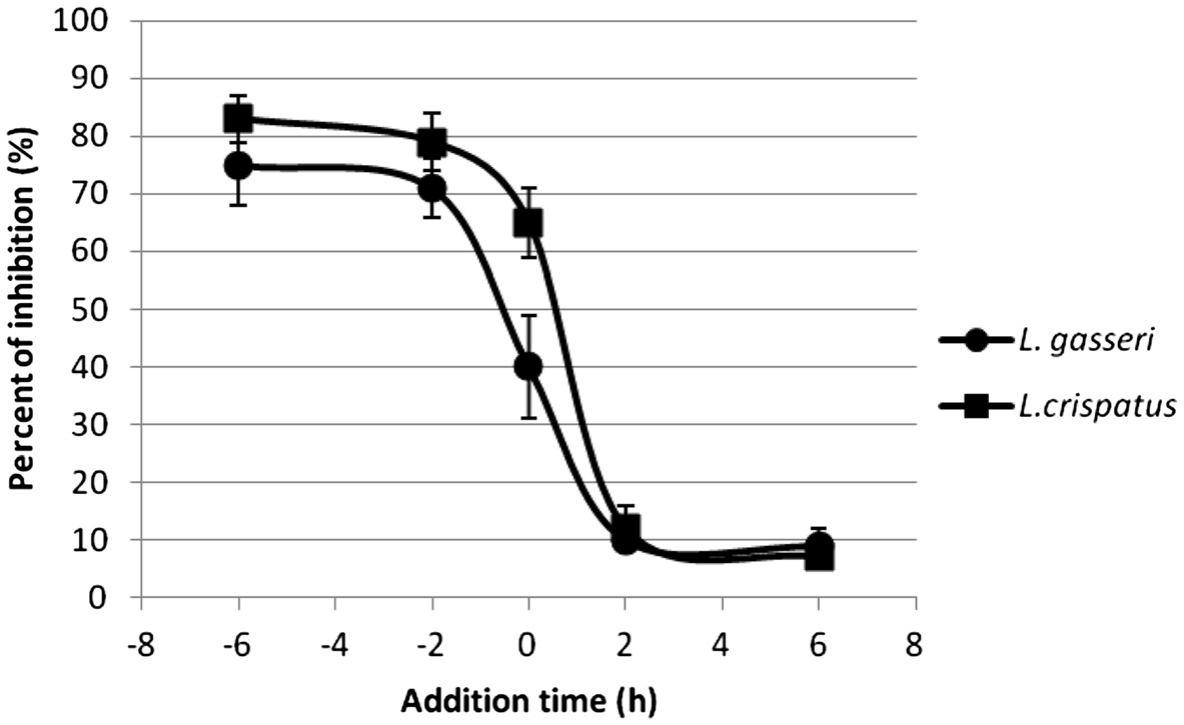
**The inhibitory effect of *L. gasseri *and *L. crispatus *culture supernatants against different stages of HSV-2 infection.** Culture supernatants (20%v/v) were added at different periods (before, during, and after) of HSV infection. The cells were washed twice by DMEM to eliminate lactobacilli supernatants prior to the inoculation of virions for pre-infection (−6, −2 and 0 h) groups.

The culture supernatants of *L. gasseri* and *L. crispatus* (20%) were added at different intervals (before, during, and after) of HSV infection. The results showed that *L. gasseri* and *L. crispatus* CS restrain HSV infection when added −6 and -2hs before virion inoculation by 75 and 83%, respectively (Figure 2). However, the inhibitory rate declined to 40 and 65% or less when *L. gasseri* and *L.crispatus* CSs and virions were simultaneously added. This data indicated that the lactobacilli supernatants affect the initial stage of HSV-2 infection. The lactobacilli products may inhibit HSV through disturbing the adhesion of virions to the cells or neutralizing the viral particles. This is a fairly new proposed mode of anti-viral activity for lactobacilli which is different from held belief that lactobacilli inactivate viral particles by lowering PH and disinfectant agent secretion [27].

### The in vivo anti-HSV activity

**Figure 3:**
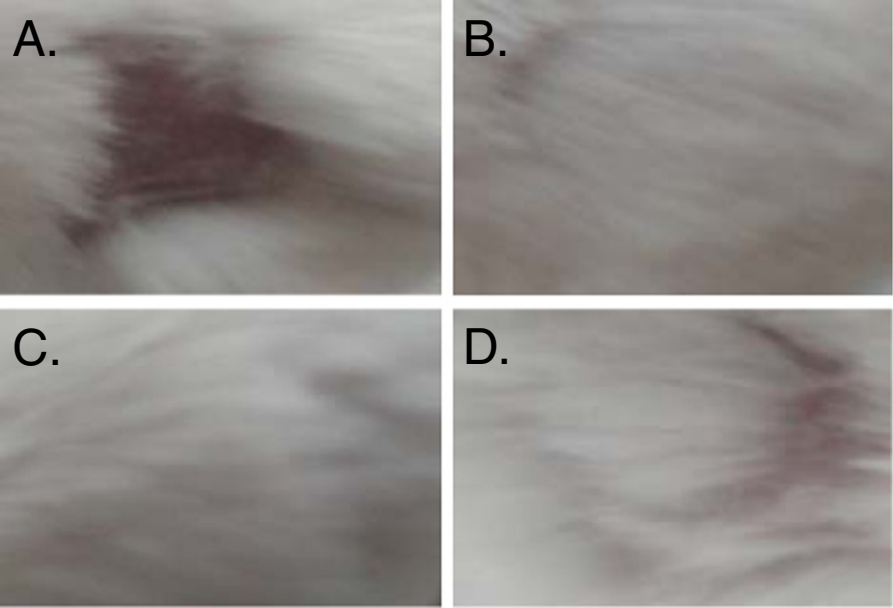
**Skin lesions 6 weeks after cutaneous infection of HSV-1 in BALB/c mice.** The shaved and scratch skin of right flank was infected with 10^4^pfu of HSV-1 and the development of skin lesions was monitored after 6 weeks. The hair loss in the site of infection was clearly seen in infected mice (**A**) in comparison with ones treated with acyclovir cream (5%) (**B**). *L. gasseri* treated mice raised normal hairs (**C**) while comparative hair loss can be seen in *L. crispatus* (**D**).

The anti-HSV-1 activity of *L. crispatus* and *L. gasseri*, strains with highest potential *in vitro* experiments, was studied in BALB/c mice model. Alive *L. gasseri* cells showed significant activity for protecting mice against HSV-1 virions infection. As it can be seen in Figure 3, *L. gasseri* treated mice raised normal hairs while comparative hair loss can be seen in *L. crispatus* and control groups. The 6.5 and 8.8 g of weight loss was just observed in mice which received *L. crispatus* or no treatment, respectively (Table 3). To prove the inhibitory activity of *L. gasseri* against HSV infection the lymphocyte cells were extracted from mice spleen and evaluated for proliferation response to HSV antigens. Spleen cells from Acyclovir and *L. gasseri* treated mice showed no response to viral antigens with stimulation index (SI) values of 1.01 and 1.05 (Table 3). These data indicates that immune cells were not exposed to HSV antigens which unequivocally show inhibition of establishment of infection. The findings of this study lead to the conclusion that *L. gasseri* cells have comparable potential for inhibition of HSV infection *in vivo* condition to Acyclovir.

**Table 3:** **The *in vivo *anti-HSV activity of lactobacilli**

**Lactobacilli strain**	**Disease progress**		**Immune response**	
	**Weight loss (g)**	**Hairless area (cm**^**2**^**)**	**Lymphocyte proliferation (OD)**	**Stimulation index (SI)**
***L. crispatus***	6.5 ± 0.4	1.7 ± 0.6	1.684 ± 0.43	1.9 ± 0.2
***L. gasseri***	0 ± 0.1	0.07 ± 0.2	0.876 ± 0.12	1.05 ± 0.3
**Negative control**	8.8 ± 0.3	4.1 ± 0.3	1.882 ± 0.62	2.1 ± 0.1
**Acyclovir**	0 ± 0.2	0.09 ± 0.01	0.793 ± 0.31	1.01 ± 0.2

## Conclusion

The results of the present study reveal the anti-HSV and HIV effect of lactobacilli by production of anti-viral molecules and other possible mechanisms. Infection was significantly reduced with no cytotoxicity if HSV and HIV were cultured in the presence of living lactobacilli cells. The presence of lactobacilli cells was not necessary for inhibitory activity since virus replication is also inhibited in the presence of neutral pH supernatants. Vaginal lactobacilli strains were able to inhibit the first stages of HSV infection. The anti-viral activity of lactobacilli cells in the midst of herpes virus binding to the target cell was strain-dependent. *L. gasseri* showed inhibitory activity against HIV virions among investigated lactobacilli in this study. Numerous mechanisms may be involved in the inhibitory effect of lactobacilli against HSV and HIV such as: interfering with the early steps of virus infection, viral particle neutralizing and secretion of compounds with the ability of blocking the intracellular events of virus replication. The *in vivo* experiment confirmed the anti-viral activity of lactobacilli in mouse models. Potent lactobacilli could be promising probiotic candidates for protection against HSV and HIV or other viruses infections transmission.

## Competing interest

The authors who have taken part in this study declare that they do not have anything to disclose regarding conflict of interest with respect to this manuscript.

## Authors’ contributions

RZ: Central contributions to conception, design and performing the experiments. Drafting and revising the manuscript. EM: Substantial contributions to conception, design and a part of experiments. SMS: Substantial contributions to conception, Analysis and interpretation of data and revising the article. ARA-S: Acquisition of data. SA-D: Acquisition of data. MHM: Corresponding author. Analysis and interpretation of data, revising the article. All authors read and approved the final manuscript.
